# The place of words and numbers in psychiatric research

**DOI:** 10.1186/1747-5341-8-18

**Published:** 2013-11-18

**Authors:** Bruno Falissard, Anne Révah, Suzanne Yang, Anne Fagot-Largeault

**Affiliations:** 1 INSERM U669, Paris, France; 2 UMR 669, Universtiy Paris-Sud and Universtiy Paris-Descartes, Paris, France; 3 APHP, Villejuif, France; 4 Centre Hospitalier Victor Dupouy, Argenteuil, France; 5 Mental Illness Research, Education and Clinical Center (MIRECC), Veterans’ Administration Pittsburgh Healthcare System, Pittsburgh, PA, USA; 6 Western Psychiatric Institute and Clinic, University of Pittsburgh School of Medicine, Pittsburgh, PA, USA; 7 Collège de France, Paris, France

**Keywords:** Qualitative methods, Quantitative methods, Mixed methods research, Hermeneutics, Induction, Abduction, Statistics

## Abstract

In recent decades, there has been widespread debate in the human and social sciences regarding the compatibility and the relative merits of quantitative and qualitative approaches in research. In psychiatry, depending on disciplines and traditions, objects of study can be represented either in words or using two types of mathematization. In the latter case, the use of mathematics in psychiatry is most often only local, as opposed to global as in the case of classical mechanics. Relationships between these objects of study can in turn be explored in three different ways: 1/ by a hermeneutic process, 2/ using statistics, the most frequent method in psychiatric research today, 3/ using equations, i.e. using mathematical relationships that are formal and deterministic. The 3 ways of representing entities (with language, locally with mathematics or globally with mathematics) and the 3 ways of expressing the relationships between entities (using hermeneutics, statistics or equations) can be combined in a cross-tabulation, and nearly all nine combinations can be described using examples. A typology of this nature may be useful in assessing which epistemological perspectives are currently dominant in a constantly evolving field such as psychiatry, and which other perspectives still need to be developed. It also contributes to undermining the overly simplistic and counterproductive beliefs that accompany the assumption of a Manichean “quantitative/qualitative” dichotomy. Systematic examination of this set of typologies could be useful in indicating new directions for future research beyond the quantitative/qualitative divide.

## Introduction

There is a longstanding, well-known tension between people who are fond of words and those who favour numbers. Among philosophers, the tradition would have it that Plato engraved “Let no-one ignorant of geometry enter here” at the entrance to his academy. For his part, Pascal in his *Pensées* contrasted “l’esprit de finesse” with “l’esprit de géométrie”: on the one hand a grasp of the world in its complexity and nuances, and on the other the clear-cut representation of reality through abstract and formal concepts that reduce it mathematically. Personal preferences in this respect may well be established early in life, arising from individual abilities, and environmental or psychological factors [1].

This tension between words and numbers has a counterpart in the academia: the fierce, ongoing struggle between quantitative and qualitative research approaches. Certain ontological and epistemological issues underpin this classic opposition; we will discuss several of them in the following section. It seems however that the debate is so passionate that political and ideological considerations can sometimes play an important role in maintaining and perpetuating a binary typology.

Psychiatry is a discipline that is particularly sensitive to this duality of words and numbers, qualities and quantities, “soft” and “hard” approaches. From the time of Pinel to the work of Freud and beyond, psychiatric researchers have relied on the narratives of patients. A narrative approach was central to the innovation known as the “moral treatment”, introduced when Pinel “broke the chains of the insane” [2]. Talking with patients - whether to support and calm them at times of acute distress or to guide them to new awareness and change - has been central to psychotherapeutic interventions over the decades and centuries. And yet today we are faced with a curious hiatus. While some clinicians continue to practice the art of narrative, researchers approach mental health through a lens that seems at odds with the daily working experience of many therapists, calling upon genetics, complex statistical models, and images of the brain. This striking contrast between “soft” clinical approaches and “hard” research has even led certain authors to caution against the “rise to prominence […] of a biological reductionist perspective” in psychiatric research [3]. Fortunately the situation is not so clear-cut. Not only is the exploration of psychosocial aspects of mental health care and systems is still quite active, but there are many emerging phenomena that are generating considerable shifts in existing categories and concepts. The development of new disciplines such as neuropsychoanalysis is one notable example. New experimental designs that can examine the first-person viewpoint in a subjective, phenomenological perspective [4] or unconscious/implicit processes that are now being investigated by cognitive neuroscientists [5] are also notable examples.

The present period of epistemological questioning requires new perspectives on how we conceptualize and categorize research methods in psychiatry. Proponents of either the qualitative or the quantitative viewpoint could retreat into their own corners, in the belief that their approach is the closest to the truth. This is logical, given the organization of research until recently, where expertise and specialization have been viewed as mastery of an ever-narrower slice of knowledge. Our proposal is the reverse: given the increasing gap between research and practice, and in the context of a tremendous expansion and diversity of methods, a new effort to integrate findings is needed.

The division of research approaches into qualitative and quantitative may well be counterproductive and ill-suited to the range of studies performed today. A more nuanced taxonomy of research methods would lay the way for future investigations by helping scientists and readers to better categorize, compare and contrast the pieces of knowledge emerging from the psychiatric literature. In the following section, we will approach the corpus of literature dealing with the basic differences between quantitative and qualitative research. A second section will be devoted to our proposal for an alternative taxonomy. Finally a discussion will conclude the paper.

### The classic opposition of qualitative and quantitative approaches in the social sciences

In recent decades, there has been widespread debate in the human and social sciences regarding the compatibility and the relative merits of quantitative and qualitative approaches in research [6]. Obviously, it is beyond the purpose of this paper to offer a comprehensive review of the literature on this vast and controversial topic. We will select two authors who are complementary in their approaches and who exemplify key positions in this debate. The first, H. Becker, in a very straightforward and traditional manner [7], sets out the fundamental differences that can be seen as contrasting qualitative and quantitative research. The second author, C. Grignon, is a noteworthy example of an alternative perspective [8], which suggests instead that there is a continuum between the qualitative and quantitative styles of thinking. The two authors work in the field of sociology, and this choice enables direct comparison within the same discipline, particularly because sociology has been so strongly affected by the present debate [9].

In a famous reference [7] H. Becker summarises in well-informed and measured terms the distinctive features of qualitative and quantitative research [7], cited in [10]. In his paper the author contrasts quantitative (here survey-based) studies on the one hand with qualitative (here ethnographic) research on the other.

Becker points out first that quantitative research relies essentially on statistical inferences. It attempts to explain phenomena (for example being addicted to illicit drugs) by contrasting groups of subjects with different traits (do addictive disorders occur with higher or lower frequency in males and females, in rural and urban areas, in people with high versus low levels of sensation-seeking?). In contrast, the qualitative researcher will try to produce a description ‘that captures as much as possible the meaning of what he/she has observed. For example, regarding people with addictive behaviours, an ethnographic qualitative study will comprehensively describe the subjects’ childhood, relationships within their families, the role of events or people met during adolescence, their social network or their relationships with the law, and so forth, pointing alternately to differences and similarities between life trajectories. Becker therefore suggests the following general characteristics, positing that qualitative research:

1) leaves more room for unanticipated data and for unplanned results, as opposed to quantitative research which relies mainly on closed questionnaires;

2) investigates in a more rigorous and complete manner the viewpoints of the persons who are studied;

3) describes people observed in the “real world”, as opposed to people in the rather artificial situation that consists in answering a questionnaire;

4) does not focus on validity (whether measurements are biased), reliability (whether measurements are reproducible) or hypothesis testing (the probability of reaching an erroneous conclusion is controlled for), but rather on accuracy (the quality of observations successfully capturing the subject’s point of view), precision (the consistency between the data collected and the research question) and scope (range of material that is studied); and finally,

5) involves subjective engagement with the material; in qualitative studies as opposed to quantitative studies, “fieldworkers cannot insulate themselves from data” [7].

These assertions could each be substantially criticized. They are however relevant if the premises of Becker’s paper are kept in mind: the author is contrasting survey-based studies with ethnographic research. It is difficult to transpose these conclusions to the field of psychiatry, where, in addition to these two methodological approaches, there are many others, such as neurobiological or cognitive studies, case studies, or computer simulations, among others. In any case, it is remarkable that even after this in-depth confrontation between qualitative and quantitative approaches Becker concludes very cautiously: “Practitioners of qualitative and quantitative methods may seem to have different philosophies of science, but they really just work in different situations and ask different questions. The politics of social science can seduce us into magnifying the differences. But it needn’t, and shouldn’t” [7].

Claude Grignon [8] reformulates the polarization between quantitative and qualitative research to produce a different answer. He attempts to identify specific characteristics that distinguish social sciences that use mathematical models and those that rely on narration. He demonstrates in a convincing manner that there is a continuum between work analysed in a formal manner as are mathematical theorems, and work presented in a purely literary manner.

Indeed, all published quantitative studies have an “introduction” and a “discussion” section, which rely heavily on natural language and thus on a qualitative perspective. On the other hand, most qualitative sociological papers do not refrain from including some basic descriptive statistics such as percentages, and they make use of diagrams, which are of a geometrical nature and can be considered formal.

In other words, on the one hand we need natural language (and thus the qualitative perspective) in studies said to be quantitative. Intuition, creativity and induction clearly rely a great deal on the suggestive power of natural language, and without any one of these three abilities there would be no possible emergence of knowledge, even in the most formalized domains such as mathematics.

On the other hand, we need formal aspects even in studies said to be qualitative, because natural language lacks internal cohesion from the point of view of logic, and extensive use of natural language alone can generate a body of knowledge where everything and its opposite can be valid and where truth relies more on rhetoric than on facts and formal relationships.

Finally for Grignon, while there is a classic opposition between qualitative and quantitative research, it arises from past disputes within academic disciplines, and from an untenable ideal of purity regarding the methods that should pervade scientific activities, a purity of which mathematics is emblematic.

The field of psychiatric research puts these oppositions, definitions and concepts to the test and challenges them. First, as suggested earlier, most papers or books published in the area of the neurosciences include a “discussion” section that tries to grasp and interpret subjective aspects of the human mind, and where even philosophical considerations are not rare, B. Spinoza being sometimes cited [10]. In short, neuroscientific publications (classically associated with the field of quantitative research) need natural language, and natural language deployed in its most sophisticated and complex forms. Conversely, let us consider the case of psychoanalysis (more closely associated with qualitative research). The numerous and well-known case studies written by Freud and his successors have led to the emergence of a particularly creative field of study of the human mind. While some authors observe possible stagnation today [11,12], this is perhaps a reflection of the lack of internal cohesion of a principally literary corpus, and of the hegemony of some rhetorical positions within the field, which can resemble those described in sociology by Grignon. Closer examination of Freud’s early work in neuroscience suggests that divergence between his work and that of biological psychiatry was an artefact of historical development that is ripe for revision [13].

### Moving beyond the quantitative and qualitative: an alternative taxonomy

From the previous section we can note that quantitative and qualitative research have been distinguished on the basis of several aspects. Among these aspects, we will focus now on two : (1) the data set, which can consist of “languaged data” [14] (sentences, paragraphs or even longer pieces of text), or coded data (categorical or quantitative variables)); and (2) the data analysis, for instance the use of equations, statistical software or resorting to a hermeneutic approach.

We will favour a categorical taxonomy in order to clarify the conceptual and epistemological differences between qualitative and quantitative approaches. This taxonomy is based on the observation that among quantitative disciplines (and this is also true for qualitative disciplines) there are differences that are so great that the quan/qual duality is perhaps only marginally relevant. For instance, is it possible to conflate classical mechanics and epidemiology simply because both deal with numbers? In classical mechanics, reality is mapped onto the purely formal set R^3^ (i.e. a point mass is characterized by three coordinates x, y and z). In epidemiology, statistics are extensively used, but to identify relationships between variables like “social status” and “depression”, which are provisional constructs defined by convention and at the very least not formal at all in the philosophical sense. If one now considers molecular biology, the use of mathematics in this discipline is limited, but even so it cannot be considered to be a qualitative discipline.

At this point, in order to proceed further, we need to define more explicitly what we mean by “mathematics”. Unfortunately, there is no consensual definition of this word. The Encyclopaedia Britannica [15] proposes “the science of structure, order, and relation that has evolved from elemental practices of counting, measuring, and describing the shapes of objects”, while a more specialized source, *Wolfram Mathworld*[16] suggests: “Mathematics is a broad-ranging field of study in which the properties and interactions of idealized objects are examined”. In short, we will consider that mathematics basically examines idealized objects (most often numbers and shapes), which can be structured and related one to the other. It is notable that the elements of natural language (words) can also be structured (into sentences, etc.) and related one to another (either via syntax or via a paradigm, as in structural linguistics [17]). But words are not idealized objects; they depend on both culture and physical reality [18] as well as on history.

As suggested above, our taxonomy is based on (i) how the objects are represented in the dataset and (ii) how the relationships between these objects are expressed in the data analysis process.

(i) Objects or parameters can be represented either by words or by numbers, and this traditionally underpins the opposition between qualitative and quantitative sciences. In classical mechanics, objects are represented and fully accounted for by the coordinates of their centre of gravity, their mass, moment of inertia, etc., which are all numerical. Conversely, in the work of Freud, patients are described in a literary style, without any recourse to numbers, although symbols are sometimes used. In psychiatric research things are however not so clear-cut. In epidemiology, in brain imaging studies, some patient characteristics can be measured (for instance a depression score, a cerebral blood flow in the prefrontal cortex, etc.), but there is no claim at all that the subject as a whole is represented and fully accounted for by these numbers. On the contrary, most scientists *do* acknowledge that the vast majority of a patient’s characteristics are unknown and even unconceptualized. In a situation of this kind, where the objects are coded numerically in a data set, but where this code provides only a weak representation of the object itself, we will describe the discipline as locally or partially mathematized. This can be compared to physics, which is globally and comprehensively mathematized, and contrasts with Freud’s psychoanalysis which is not mathematized at all in the sense defined above.

(ii) Concerning the representation of relationships between objects as expressed in the data analysis process, the situation is similar. In classical mechanics, relationships are represented with equations. Even if the law of gravity can be expressed in words: “every point mass in the universe attracts every other point mass with a force that is directly proportional to the product of their masses and inversely proportional to the square of the distance between them” [19], this law translates a strictly deterministic mathematical equation. In the work of Freud, there are also laws of this kind: “Sexual life does not begin only at puberty, but starts with plain manifestations soon after birth” [20] (p. 23). But, of course, there is no possible mathematical equation of any sort, and, furthermore, the assertion certainly has not the same strength. The law of gravity is considered to be true (even if there are experiments that refute it [21], but that is another story). It is assumed to be true everywhere on the earth, today as it was 10 million years ago or as it will be in 10 million years’ time. Conversely, the assertion made by Freud is based on clinical observations and a hermeneutic process [22]; it is inductive by nature and might no longer be relevant in certain societies or at some other time.

The opposition between the use of equations and hermeneutics to characterise laws and relationships is thus another key feature opposing quantitative and qualitative research. There are however many disciplines relevant to psychiatric research that do not readily fit this dichotomy. To focus on the fields of epidemiology and brain imaging, even if these disciplines examine relationships that are based on numerical considerations, they do not develop laws based on deterministic equations. Consider for instance the two statements: “heavy use of cannabis before 15 increases by 4 the risk of having a schizophreniform disorder at 26” [23] or “patients with depression have a statistically significant 19% smaller left hippocampal volume than comparison subjects” [24]. These relationships are numerical expressions of associations that have been observed within a given sample of subjects, to test whether these observations are likely to be applicable on average to an underlying population. In other words, these relationships are of a statistical nature and do not claim to incorporate any real objects into a purely mathematical representation that will hold universally true in other circumstances. Relationships between objects can thus be established from equations, from a hermeneutic process, and also from statistics.

In summary, we propose that scientific knowledge, especially when viewed in the light of psychiatric research, is composed of the following categories: (i) *objects* of different types (ii) patterns of *relationships*.

Scientific objects can be represented in three main modes:

1. In a purely literary manner (i.e. the objects are represented by words, in a text, for example the work of Freud).

2. Locally using mathematics (i.e. only some facets of the objects are represented by numbers, the others are represented by words, often implicitly. Epidemiology is an example here).

3. Globally using mathematics (i.e. the objects are comprehensively represented by numbers or shapes. It is possible to name them, to talk about them, but their nature is mathematical. Classical physics or quantum mechanics are of this nature).

The relationships between these objects are established:

A. using a hermeneutic process (this is the case for most of the work by Freud).

B. from statistical analyses (this is the case when it is said that height and weight are positively correlated: if two persons are randomly selected and if one is taller, then the probability that he/she is also heavier is greater than 0.5).

C. from deterministic equations (the Archimedes principle is of this nature).

These two categorisations can be cross-tabulated in a 3 by 3 grid (see Table 1).

**Table 1:** Proposal for a taxonomy of psychiatric research

		**Relationships between observations are obtained from**		
		**A) Hermeneutics**	**B) Statistics**	**C) Equations**
**Symbolisation of observations**	*1) Literary*	Psychoanalysis, most transcultural psychiatric studies	Competerized textual analysis	Many neurobiological mechanisms
	*2) Locally mathematical*	ABAB designs for evaluation of CBT	Epidemiology, Cognitive neuroscience, Brain imaging	Pharmacokinetics of psychotropic drugs, Lacan’s “graphe du désir”
	*3) Globally mathematical*	Spin glass model of brain functioning	None at the moment in psychiatric research [thermodynamics]	None at the moment in psychiatric research [most of physics]

Objects can be represented globally with mathematics, locally with mathematics or without mathematics. Relationships between these objects can be obtained from a hermeneutic process, from equations or from statistical analyses.

We will now focus on describing each of the 9 corresponding cells.

(1 A) Literary × hermeneutics:

in psychiatric research, there is a long tradition of literary work that is based on a hermeneutic perspective. We have already cited the previous work by Freud. This approach is still active today in the field of psychoanalysis, as well as in ethnopsychiatry or in the history of psychiatry. It is however notable that the main general psychiatric journals like *European Psychiatry* or *The American Journal of Psychiatry* do not usually publish studies of this kind. Although the journals may include historical or personal vignettes and clinical cases, this type of work does not form the core of publications defined today as research.

(1 B) Literary × statistics:

text mining, or text analytics, refers to the process of deriving information from text using statistical tools. Text mining has been used for the authorship analysis of Shakespeare’s work [25]. It is sometimes used in psychiatric research [26]. For example, in a recent paper by some of the present authors, computer-assisted linguistic analysis was used to enhance a traditional qualitative analysis of the impact of long-term incarceration, including subjects with severe mental illness [27].

(1 C) Literary × equations:

many claims in neurobiology can in fact be based on this approach. Consider for instance the assertion: “Fluoxetine is a selective serotonin reuptake inhibitor”. This assertion is made up of words and cannot be translated into any mathematical form; at the same time this is not a pure narrative, nor is it obtained from a hermeneutic process. Neurobiologists have shown repeatedly using non-literary techniques that Fluoxetine is a potent inhibitor of the neuronal reuptake pump for serotonin. Fluoxetine also inhibits the neuronal reuptake of norepinephrine and dopamine, but the binding affinity is much weaker compared to serotonin [28]. Over time, neurobiologists have mapped their experimental dataset into a structured set of words and definitions, and from the results observed with Fluoxetine it is said that it “is a selective serotonin reuptake inhibitor”, a word formula that has properties like a mathematical equation. In principle, this is not categorically true (because in particular it is not totally selective). But inside the structured, monosemous language of neurobiology it is, and remains, a definition or label that is used for experiments that attain consensus and are reproducible. This is the reason why we will consider here that neurobiology uses equations and not a hermeneutic approach.

(2 A) Locally mathematized × hermeneutics:

In the evaluation of behavioural therapies, single-subject experimental designs have been used for a long time [29]. If A and B are two therapeutic conditions, “AB” designs refer to experimental situations where a patient is given all A treatments first, then all B treatments. These designs can straightforwardly be extended to “ABA” or “ABAB” designs. At each time, an outcome is measured numerically, so that a graph can show the patient’s evolution across time according to the treatment received, A or B. While it is possible to statistically test the differences in outcome between A and B, it is fairly complex [29] and many researchers prefer to rely on visual inspection of the graph [30]. An inference based on the visual inspection of a pattern on a graph is basically inductive and is thus closer to some kinds of hermeneutics than to equations or statistical analyses.

(2 B) Locally mathematized × statistics:

as mentioned above, this is the case of most research published in general psychiatric journals today. Epidemiology, brain imaging, cognitive studies, and randomised controlled trials all use a given set of variables measured in subjects, and the objective of most measurements is to find a scientifically relevant and statistically significant relationship between two or more of these variables.

(2 C) Locally mathematised × equations:

in practice, biological psychiatrists have to consider the half-life of psychotropic medications (the time required for the concentration of the drug in the body to reduce to one half). Pharmacologists have shown for instance that the “half-life of Fluoxetine and its active metabolite Norfluoxetine is 7 to 15 days” [31]. This assertion is clearly related to a locally mathematized context and, using the same argument as developed in [1. A.] concerning neurobiology, we are here in a functionalist (i.e. dealing with equations) rather than a statistical perspective. At first glance this proposal could appear curious because the half-life of a drug is determined by pharmacokinetic experiments, and pharmacokinetics *does* use statistics. However, once the half-life of Fluoxetine has been established in a scientific paper, it is considered as a universal numerical constant (even if it is presented as an interval such as 7–15 days), which becomes a key parameter of the ideal exponential evolution of the concentration of Fluoxetine in blood across time. In a very different manner, the psychoanalytic structuralist movement led by Lacan tried to represent some cornerstones of psychodynamic psychopathology using mathematical formulae or graphs [32]. Here also, this corresponds to a locally mathematized and functionalist way of representing objects and relationships (functionalist because it relies on mathematical representations of abstract elements and logical inference based on the principles of functioning in Lacan’s symbolic register, rather than inductive reasoning by observation of patterns in empirical data [32]).

(3 A) Globally mathematized × hermeneutics:

a globally mathematized approach to psychopathology is likely to appear to most specialists as pure science fiction. Curiously one such approach does exist and was developed in the early 1980s. At that time, theoretical biophysicists developed so-called “spin glass models” of brain functioning, where the human thinking process is represented as a marble rolling in a landscape [33]. The depressions in this landscape correspond to learned configurations known as “attractors”. This is indeed a fully formalized model of human thought and it has been recently examined as a heuristic device in relation to the psychoanalytic and psychiatric phenomenological perspectives [13]. This approach is however very preliminary and should be seen for the moment only as a metaphor, basically relying on a hermeneutic perspective. In the same context, catastrophe theory is also potentially relevant. Catastrophe theory is an attempt to think in geometrical and topological terms about problems as different as biological regulation or syntactic structure [34]. It has also been used to model human behaviours and psychopathology, anorexia nervosa in particular [35]. This too remains a theoretical device or metaphor that is useful for conceptualization and interpretation, and is thus a hermeneutic use of generalized mathematical representations.

(3 B) Globally mathematized × statistics:

To our knowledge there is no theory of this kind at the moment in the field of psychiatric research considered in the broadest sense. In physics, quantum mechanics or statistical thermodynamics exemplify this perspective as an ideal. However, this is not so obvious in fact, particularly regarding quantum mechanics, which is not statistical at a pragmatic, methodological level. Instead, it is probabilistic in its essence at a conceptual level and could thus be considered as globally mathematized and functionalist [36].

(3 C) Globally mathematized × equations:

At the present time, there is no perspective of this kind in the field of psychiatric research. It is however the approach typically used in physics since, in particular, classical mechanics and the theory of relativity rely upon it. Here, all objects are represented by elements of a geometric space R^3^ or R^4^ and the interactions between these objects are expressed using formulae and equations that are not statistical and probabilistic but deterministic. Newton’s law of universal gravitation is a typical example of this.

## Discussion

In recent decades, there has been widespread debate regarding a questionable compatibility [37] and the relative merits of quantitative and qualitative approaches for research in several academic fields [38]. Many key papers have been written on this topic [6] and most of them discuss, challenge and contrast the features that tend to support the choice of quantitative, qualitative or even mixed approaches in particular research questions. These papers draw broadly upon philosophical works from Aristotle to David Hume, Immanuel Kant, August Comte, Karl Popper, Ludwig Wittgenstein and many others. In contrast, the present paper focuses on two very basic features to examine a taxonomy of research in psychiatry: the mathematical or non-mathematical characteristics of the dataset, and the mathematical or non-mathematical ways that data are analyzed.

A dataset consists traditionally either of numerically coded data or of “languaged” data. A numerically coded dataset, i.e. a dataset that comprises categorical or quantitative variables, can however represent two different kinds of “reality”: one is locally mathematized while the other is globally mathematized. In psychiatric research, the use of mathematics is most often only local, to measure a limited characteristic of the phenomenon, as opposed to classical mechanics where the entire phenomenon is posited to be comprehensively mathematized and captured in the measurement. More precisely, with the exception of spin glass models, there is no global mathematical picture of the thinking subject. Our current mathematization in psychiatry is limited to certain facets of a given phenomenon that are too distant from one another to be merged into a single, global construction.

Whether psychiatric entities are represented by words or by mathematical objects, their relationships can also be established and expressed in different ways: using a hermeneutic process, using statistics or using equations. Equations capture formal and deterministic relationships. This does not mean that people actually *believe* that a so-called “reality” obeys these formal laws; it is just the way relationships are expressed and used in the development of further knowledge. The statistical manner of representing relationships is the most frequently used in psychiatric research. Here, samples of subjects are observed and statistical computations are used to test the hypothesis that some of the variables measured are dependent on and significantly related to each other. There are no deterministic laws here but rather certain relationships observed “on average”. Alternatively, it happens that relationships are posited and obtained from inductions and abductions derived from qualitative descriptions and this methodological approach is sometimes designated as “methodical hermeneutics” [39].

Some could argue at this point that statistics are a branch of mathematics and that obviously equations are very common in this discipline (linear regression models, often used in psychiatric research, are indeed equations). The distinction between the two categories “statistics” and “equations” (B. and C.) could thus be highly questionable. The difference is that statistical procedures do not inherently provide formal, universal and deterministic relationships. In short, deterministic equations provide relationships that are supposed to be true in all instances, for everyone, while most statistical relationships are only true for an average subject, who does not exist in practice. The conceptual difference is immense and it may clarify and soften the classic opposition between the nomothetic and idiographic perspectives. It is indeed traditional to contrast “knowledge construction that emphasizes the general (nomothetic) and that which focuses on the particular (idiographic)” [40,41]; in practice, the general is associated most often with the natural sciences (considered to be fundamentally quantitative, with emphasis on reproducible regularities) while the particular is more a prerogative of social and human sciences (considered to be more accessible to qualitative approaches, given specific cultural and social contexts). This historical dichotomy of the “general/nomothetic/quantitative” versus the “particular/idiographic/qualitative” is however more and more in doubt [41,42] and the tripartite division proposed here (equations, statistics, hermeneutics) offers the potential to shed new light on the debate.

The 3 ways of representing entities and the 3 ways of expressing relationships can be combined, and nearly all nine combinations can be observed in some way in current research. This highlights the artificial basis for a binary contrast between words and numbers, between literary expression and mathematics, between qualitative and quantitative approaches in psychiatric research. There is an emerging multiplicity of different ways to conceptualize and design psychiatric research. The typology developed here is more nuanced than the traditional dichotomy “QUANTI/QUALI”. Dismantling this opposition, which appears to have outlived its usefulness, could lead to other ways of thinking about methods that are more precise and relevant to long-term goals in the field.

There is considerable potential value in developing a non-binary typology of this sort, to identify which perspectives are dominant at a given moment, and which should be further explored. It even affords the possibility of indicating entirely new directions, and prospects for using combinations of methods to refine our approach to critical questions. Most important, it may help us to overcome the repetition of a worn-out and overly simplified opposition, where scientists and clinicians alike come to believe either that the use of quantitative methods brings us closer to the truth, that they are more objective, or conversely that they cannot possibly access the realities of mental life.

Beliefs such as these have had impact at a policy level, using the qualitative-quantitative divide to set priorities, with far-reaching consequences. The 2001 “No Child Left Behind Act” to improve the education of children in U.S. public schools explicitly considered quantitative and experimental studies as the gold standard for program evaluation, selection and revision. Some qualitative researchers [42] reacted to this stance by stating that privileging quantitative evaluation and standardized testing was an ideological position, using the term “methodological fundamentalism” to describe the monolithic endorsement of one type of research method. In contrast, a short time later, the U.S. Government Accounting Office (GAO) issued a policy statement supporting the use of ethnography in program development and evaluation [43]. More directly related to mental health and psychiatry, in 2005, in response to a lively controversy in France concerning the relevance of evidence-based practices for the evaluation of psychotherapies, the French Minister of Health stated publicly that “mental suffering can be neither measured nor evaluated [quantitatively]” [44] and publicly took a position against imposing evidence-based practices in this field. The development of typologies that are more nuanced than the Manichean contrasting of quantitative versus qualitative could thus be timely for developing less polemical positions, at individual, academic or societal levels and in key policy debates.

In addition, going beyond the qualitative-quantitative divide could foster increased awareness of the potential influence of the investigator’s perspective on the phenomenon under study. In the preface to their book, Denzin and Lincoln emphasise the crucial position of the observer in qualitative studies: “Qualitative research is a situated activity that locates the observer in the world” [45]. This also holds for quantitative studies, where neutrality is not guaranteed. The taxonomy introduced in this paper shows that the choice to use mathematics or not is fundamentally independent from the degree of neutrality of the observer: mathematics is not one but many different means that can be used to describe the world. The role of the observer is perhaps a matter of ‘habitus’ for researchers. Qualitative researchers have openly organized their practice around an awareness of how they are situated in relation to the object of study, and influence it, while quantitative researchers most often deny the issue, with the exception of quantum mechanics. Our analysis of the longstanding opposition and perceived antagonism between qualitative and quantitative approaches suggests that differing degrees of mathematical representation each involve subjective and heuristic choices that impact the results and their interpretation.

Finally, any immoderate preference for either the quantitative or the qualitative perspective viewed as a binary choice can also affect clinical practice. A systematic preference for qualitative approaches among individual practitioners could lead to discounting of the large body of therapeutic knowledge derived, for example, from randomized controlled trials published in the scientific literature. Conversely, over-reliance on the use of quantitative measurements in day-to-day clinical practice has been thought to potentially contribute to negative patient perceptions of outcomes [46]. One interpretation of this finding is that the use of a quantitative measurement could facilitate the disengagement of the physician from the doctor-patient relationship and from the patient’s psychological suffering. If clinicians are schooled exclusively in research results where patients are described only by scores, cerebral blood flow or reaction times, their understanding of the subjective experience of a mental disorder may become secondary and perhaps of diminished importance. There would then be a risk that the empathic stance of the clinician may wane progressively and be replaced by a pseudo-objective, distanced attitude. By differentiating the ways that objects of study are constituted in words, numbers or symbols and by distinguishing modes of articulating the relationships between them, whether mathematical or non-mathematical, we may identify alternative ways to represent patient experience. With greater awareness of a taxonomy of methods, we may be more able to conceptualize clearly the different scientific languages that can be used and combined. A more precise strategy could then emerge to systematically renew the link between research with the language of clinical practice.

## Competing interests

The authors declare that they have no competing interests.

## Authors’ contribution

BF wrote the first version of the paper that has then been edited by all the authors. All the authors participated to the research. All authors read and approved the final manuscript.
